# The Addition of Transdermal Delivery of Neostigmine and Glycopyrrolate by Iontophoresis to Thrice Weekly Bowel Care in Persons with Spinal Cord Injury: A Pilot Study

**DOI:** 10.3390/jcm10051135

**Published:** 2021-03-08

**Authors:** William A. Bauman, Anton Sabiev, Shahzad Shallwani, Ann M. Spungen, Christopher M. Cirnigliaro, Mark A. Korsten

**Affiliations:** 1 Veterans Affairs Rehabilitation Research and Development Service’s National Center for the Medical Consequences of Spinal Cord Injury, James J. Peters Veterans Affairs Medical Center, Bronx, NY 10468, USA; anton.sabiev@va.gov (A.S.); ann.spungen@va.gov (A.M.S.); christopher.cirnigliaro@va.gov (C.M.C.); mark.korsten@va.gov (M.A.K.); 2 Medical Service, James J. Peters Veterans Affairs Medical Center, Bronx, NY 10468, USA; shahzad.shallwani@va.gov; 3 Departments of Medicine and Rehabilitation and Human Performance, The Icahn School of Medicine at Mount Sinai, New York, NY 10029, USA

**Keywords:** spinal cord injury, neurogenic bowel, difficulty with evacuation, neostigmine, glycopyrrolate, iontophoresis

## Abstract

Persons with spinal cord injury (SCI) have neurogenic bowel disorders characterized by difficulty with evacuation (DWE), fecal incontinence, and discoordination of defecation. Six medically stable in-patients with SCI with a mean age of 57 ± 10 years (range: 39–66 years) and time since injury of 18 ± 17 years (range: 3–47 years) were investigated. Standard of care (SOC) for bowel care was followed by two weeks of SOC plus neostigmine (0.07 mg/kg) and glycopyrrolate (0.014 mg/kg) administered transcutaneously by iontophoresis thrice weekly for two weeks while patients continued to receive SOC. The primary endpoint was time to bowel evacuation. Body weights and abdominal radiographs were obtained. Ten questions related to bowel function and the Treatment Satisfaction Questionnaire for Medication were acquired after each arm. Bowel evacuation time decreased after the dual drug intervention arm (106.9 ± 68.4 vs. 40.8 ± 19.6 min; *p* < 0.0001). Body weight decreased (2.78 ± 0.98 kg; *p* < 0.0001), a finding confirmed on abdominal radiograph. Both questionnaires demonstrated improvement after the dual drug intervention arm. No major adverse events occurred. The addition of neostigmine and glycopyrrolate by transcutaneous administration to SOC for bowel care in persons with SCI and DWE resulted in the safe, effective, and predictable bowel evacuation with subjective improvement in bowel care.

## 1. Introduction

Persons with motor-complete spinal cord injury (SCI), as well as the majority of persons with motor-incomplete spinal cord lesions, have bowel dysfunction, which is a condition that is characterized by difficulty with evacuation (DWE), fecal incontinence, and discoordination of defecation due to dyssynergia between colonic motility and the external anal tone [1]. The clinician prescribing a bowel care regimen for the patient with SCI strives to lessen morbidity by maintaining continence and, if possible, providing the ability to defecate at will. This is frequently accomplished by identifying an individualized bowel regimen by empiric trial and error that may include diet, stool softeners, enemas, and scheduling of bowel care. Despite these approaches, bowel care is frequently unpredictable, time consuming, and often unsatisfactory for those with SCI, adversely affecting quality of life [2,3,4].

Our group has demonstrated the safety and efficacy of the intravenous and intramuscular administration of neostigmine and glycopyrrolate to induce a safe and predictable bowel evacuation [5,6]. However, the practical utility of the parenteral administration of medication for bowel care is limited due to personal, practical, and medical reasons. As such, to be of any clinical value for routine bowel care in patients with SCI, an alternative mode of administration for this dual drug combination needed to be identified.

To date, the transcutaneous administration of drugs by iontophoresis has been used somewhat sparingly in clinical medicine, and its application has been predominantly to target the delivery of agents to local tissues [7]. Iontophoresis is pain-free, does not require adherence to aseptic technique, allows for low-risk self-administration and, obviously, obviates the role for needles and injection. In addition, a potential therapeutic advantage of transdermal administration of some drugs is their direct delivery into the systemic circulation, avoiding first-pass hepatic metabolism. The application of this methodology may have practical utility in the treatment of certain conditions that require repetitive systemic administration in the home setting, such as that for bowel care in those with SCI or other conditions associated with neurogenic bowel disorders, as well as for those in the general population without a diagnosis of neurogenic bowel but associated with DWE. Our group has reported that a single transcutaneous administration of neostigmine and glycopyrrolate stimulates a predictable bowel movement without adverse local or systemic events [8]. The question remains, and is the subject of the work presented herein, as to whether the addition of this dual drug approach by transcutaneous route to standard of care (SOC) for the bowel management confers any clinical or patient-reported benefits over that of SOC alone.

## 2. Subjects and Methods

Six medically stable male patients with chronic SCI (>1 year) and DWE (bowel evacuation time > 60 min) with varying degrees of completeness of lesion who were hospitalized on the Spinal Cord Injury Service of the James J. Peters Veterans Affairs Medical Center (JJP VAMC) were recruited for study participation. A history of cardiac or pulmonary disease, uncontrolled hypertension, current infection, and/or pregnancy excluded patients from study participation. Each patient who was recruited for study participation continued to receive his individualized bowel care regimen thrice weekly during the course of the study. The study was performed in agreement with good Clinical Practice guidelines and according to the guidelines of the Declaration of Helsinki. The protocol was approved by the Institutional Review Board of the JJP VAMC. Written informed consent was obtained from each participant. The clinical trial was registered with ClinicalTrials.gov (NCT04671030).

The study consisted of two arms: (1) SOC for bowel three times a week for one week or (2) SOC plus the dual drug combination of neostigmine 0.07 mg/kg and glycopyrrolate 0.014 mg/kg administered transcutaneously by iontophoresis three times a week for two weeks. Bowel care was provided on alternate days, either Monday-Wednesday-Friday or Tuesday-Thursday-Saturday. Baseline and follow-up evaluations were performed before and after each arm of the study and included body weight and an anteroposterior abdominal radiograph. The abdominal radiograph was performed prior to treatment and on the last day of the 2-week treatment period. The images were read for the level of fecal impaction by the radiologists who were blinded as to the phase of the protocol. Using a list of questions from Lynch et al. [9], ten questions were selected and assigned a response score on a five-point scale with a “1” (best) to “5” (worst) response score; this survey was entitled the Ten Question Bowel Survey (10Q), which is not a validated survey vehicle. Rather than only questions 1 to 3 and question 14 being scored on a 7-point scale, the Treatment Satisfaction Questionnaire for Medications [10] was adapted by having all questions scored on a 7-point scale, except question 4 which had a dichotomous answer (“yes” or “no”); as such, the Treatment Satisfaction Questionnaire for Medications, as adapted for this study, is not a validated survey vehicle. The Treatment Satisfaction Questionnaire for Medications and the 10Q Survey were performed at the end of each study arm. Time to stool evacuation was averaged for the bowel care sessions of each subject for each study arm and then averaged for all subjects for that arm of the study. To capture potential adverse events after administration of the agents, blood pressure, heart rate and pulse oximetry were monitored throughout each bowel care session with assessments performed every five minutes for the initial 60 min and then at 90 min.

The skin was prepared at the sites of placement for the anode and cathode electrodes prior to placing the iontophoresis patches. At the placement site for the anode patch, the skin of the anterior thigh was cleaned with 70% alcohol preparation pads and then sprayed with 20% benzocaine followed by epilation and the application of 0.2% sodium lauryl sulfate in deionized water. At the placement site for the cathode patch, which was approximately 4 to 6 inches distant from the anode patch electrode on the lower extremity, the skin was cleaned with 70% alcohol preparation pads. The anode patch was loaded with neostigmine and glycopyrrolate mixed in distilled water in concentrations previously described [8]. The cathode patch was loaded with 0.5 mL 0.9% normal saline with 1.0% citric acid. The electrodes were connected to Dynatron^®^ iBox™ (Salt Lake City, UT, USA) which delivered an electric current (4.0 mA/min) that was insensible to the subject and applied for 20 min.

### Statistical Analyses

The results are expressed as the group mean plus or minus standard deviation (SD). For the time to bowel evacuation and change in body weight, a two-tailed paired *t*-tests was performed. The survey scores are presented for descriptive purposes only for the Ten Question Bowel Survey and Treatment Satisfaction Survey for Medications, which were performed following each arm of the study. An *a priori* level of significance was set at *p* ≤ 0.05. Statistical analyses were completed using IBM SPSS Statistics (IBM, version 27 for Windows, Armonk, NY, USA) and graphs were generated by Prism (GraphPad Software, version 9.0 for Windows, San Diego, CA, USA).

## 3. Results

The mean age of the male subjects was 57 ± 10 years (range: 39–66 years) with a mean duration since SCI of 18 ± 17 years (range: 3–47 years) (Table 1). Three patients had a complete motor lesion with partial sensory sparing (International Standards for Neurological Classification of Spinal Cord Injury (ISNCSCI) grade B) and three patients had motor-incomplete lesions with partial sensory (ISNCSCI grade C and D) (Table 1); five of six subjects had spinal cord lesions above thoracic level-6.

**Figure 1:**
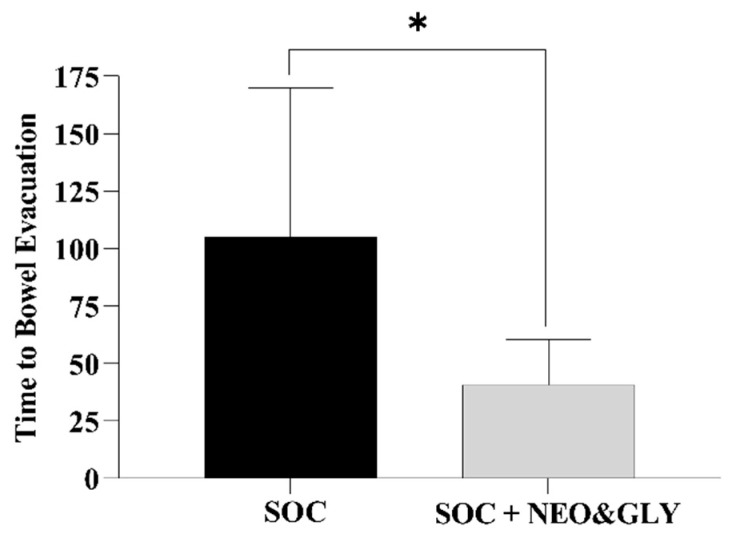
Comparison of the time to bowel evacuation between the standard of bowel care or standard of bowel care plus neostigmine and glypyrrolate arms of the study. SOC = standard of care; NEO = neostigmine; GLY = glycopyrrolate. * *p* < 0.0001.

**Figure 2:**
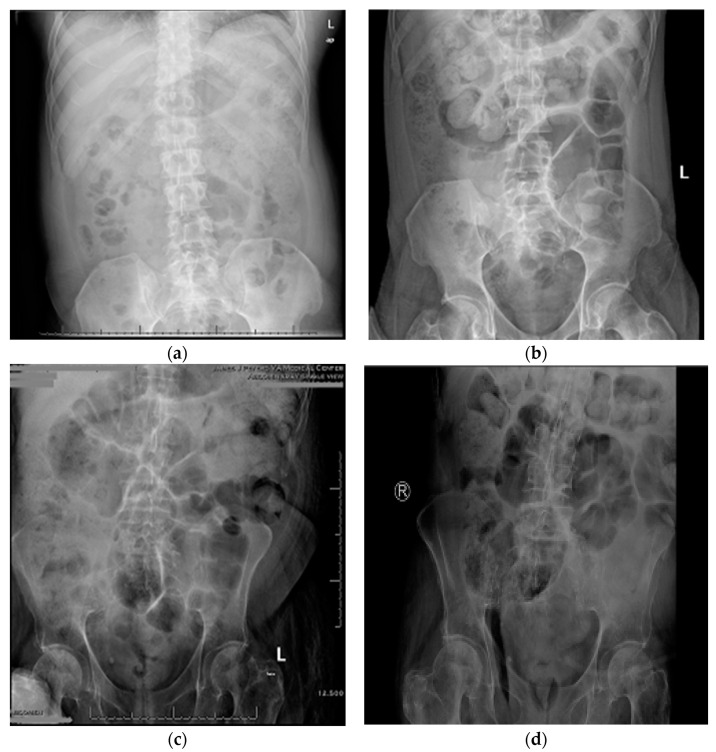
Representative qualitative measure of stool burden on abdominal radiograph after standard of care or standard of care plus neostigmine and glypyrrolate. Fecal burden: (**a**) marked stool throughout the colon, (**b**) moderate stool in the cecum; (**c**) moderate stool in the transverse and left colon, (**d**) moderate stool in the cecum. Loss of body weight after two weeks of standard of bowel care and the dual drug combination: (**a**,**b**), −4.4 kg; (**c**,**d**), −2.6 kg.

**Figure 3:**
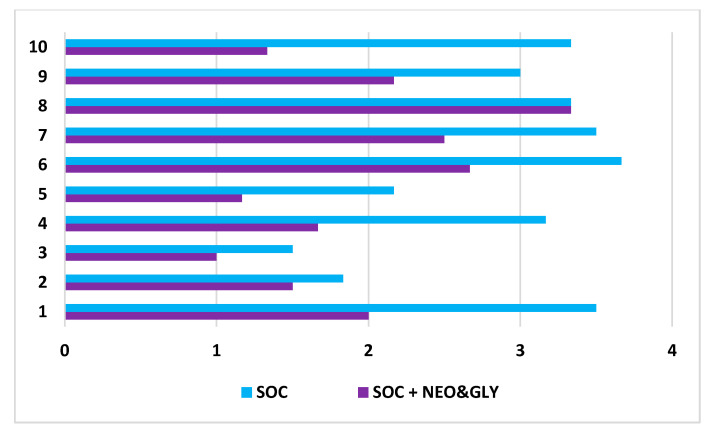
Findings of the ten question bowel survey after standard of care or standard of care plus neostigmine and glypyrrolate. Abscissa axis: A score of “1” represent fully satisfied or the best response; a score of “5” represents fully dissatisified or the worse response score. Ordinate axis labels: 1. Satisfaction with overall bowel management program during the past month; 2. Bowel control over the past month; Questions 3 to 7, 9, and 10 are asked during the past 7 days: 3. Bowel control over; 4. Use of enemas for bowel control; 5. Use of laxatives; 6. Digital stimulation; 7. Number of bowel movements (1: 7 times or more, 2: 5–6 times, 3: 3–4 times, 4: 1–2 times, 5: none); 8. Average time spent to have a bowel evacuation per bowel care session; 9. Total time in the past week; and 10. Discomfort rating. SOC = standard of care; SOC and NEO + GLY = standard of care plus neostigmine and glycopyrrolate.

**Figure 4:**
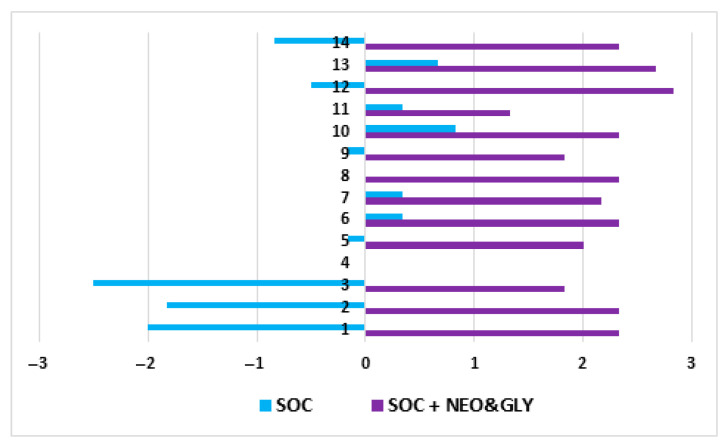
Findings of the Treatment Satisfaction Survey after Standard of Care or after Standard of Care plus Neostigmine and Glypyrrolate. The treatment satisfaction questionnaire for medications [10] was adapted by having all questions scored on a 7-point scale, except question 4 which had a dichotomous answer Abscissa: (−3) Extremely dissatisfied, (−2) Dissatisfied, (−1) Mildly dissatisfied, (0) Ambivalent, (1) Mildly Satisfied, (2) Satisfied, (3) Extremely Satisfied. Ordinate Axis: 1. Ability of medication to treat DWE; 2. Ability of medication to relieve symptoms; 3. Delay in its effect; 4. Side-effects of SOC and NEO + GLY, 5 subjects answered “yes”, 1 subjects answered “no”; 5. How bothersome are the side-effects? 6. Side-effects and physical function and health; 7. Side-effects and mental function and health; 8. Side-effects affecting satisfaction with the medication; 9. Difficulty in use; 10. Difficulty in planning; 11. Convenience in following instructions; 12. General satisfaction with the medication; 13. How certain are you that the good things about your medication outweigh the bad things? 14. Taking all things into account, how satisfied or dissatisfied are you with this medication? SOC = standard of care; SOC and NEO + GLY = standard of care plus neostigmine and glycopyrrolate.

One-week SOC in six subjects consisted of 18 bowel care sessions, and the two-week SOC plus neostigmine and glycopyrrolate consisted of 36 bowel care sessions. At the conclusion of the SOC arm, the average length of time to complete a bowel care session was 107 ± 68 min, whereas at the termination of the SOC arm plus neostigmine and glycopyrrolate arm the average length of a bowel care session was markedly shortened to 41 ± 20 min (Figure 1; *p* < 0.0001); the difference in the length of bowel care between the control and drug-treatment arms ranged from 42 to 88 min (CI: 95%). After one-week of SOC, there was no significant change in body weight (0.33 ± 0.21 kg) and, as expected, no change in abdominal radiographic images of stool burden. In contrast, at the end of two weeks of SOC plus the dual drug-treatment, an average 2.8 ± 1.0 kg loss of body weight was observed (86 ± 25 kg vs. 83 ± 26 kg; *p* < 0.0001), with an initial 1.2 ± 1.2 kg loss of weight at the end of the first week. The values for individual weight loss after the dual drug intervention arm are provided (Table 1). The loss of body weight was confirmed on abdominal radiographs to be due to a reduction in retained stool (Figure 2). After two weeks of the dual drug treatment, the 10Q Survey showed an improvement in bowel care (Figure 3), and the Treatment Satisfaction Questionnaire for Medications revealed that the medications were well tolerated and that bowel care appeared to be improved as well (Figure 4).

No severe cardiopulmonary adverse events were observed in the dual drug treatment arm. Heart rate and blood pressure, while affected by the dual drug intervention, were well within acceptable clinical limits for a treatment protocol and were not associated with any reported symptoms attributable to these minor perturbations in vital signs. Heart rate at baseline was reduced to a nadir heart rate at 40 min after the start of the dual drug intervention (72 ± 10 beats per minute (range: 59–83) to 61 ± 9 beats per minute (range: 52–77); *p* < 0.05). Systolic blood pressure increased from 108 ± 15 to 123 ± 16 mg Hg at 40 min after the start of the dual drug intervention, which was not a significant rise in systolic blood pressure and likely represented an autonomic response to stool evacuation. Pulse oximetry values were stable throughout both arms of the study. Abdominal discomfort, or cramping, occurred in all patients, was on average 2/5 in severity and persisted for 24 ± 14 min. As appreciated, abdominal cramping was an expected effect of the dual-drug intervention and represented an increase in bowel motility being sensed by the subject. The sensation of dry mouth occurred in 4 of 36 bowel care sessions at 20 to 35 min after beginning the dual drug-treatment and persisted, on average, for 35 min. Headache of 1/10 severity, which lasted an average of 13 min, was reported twice in one subject. No episodes of autonomic dysreflexia occurred.

## 4. Discussion

The transdermal delivery of neostigmine and glycopyrrolate by iontophoresis substantially reduced the time to bowel evacuation in patients undergoing routine bowel care and resulted in a more complete stool evacuation, as determined by body weight and abdominal radiographic evidence. Subjectively, the patients showed improvement in all indicators of bowel care and were more satisfied with their bowel treatment regimens.

Individuals with complete SCI have neurogenic bowel, and most of those with incomplete SCI have varying degree of bowel dysfunction. The neurological manifestations of bowel dysfunction in those with SCI may include reduced gastrointestinal motility, loss of external anal sphincter voluntary control, and impaired anal sensation; these neurogenic bowel manifestations may result in abdominal distension, intractable constipation, prolonged defecation, fecal incontinence, and eventually hemorrhoids, rectal prolapses and perianal skin complications, all of which adversely impacts quality of life. Other than bladder problems, gastrointestinal disorders are the most common secondary complication reported in patients with SCI. In a survey of 241 individuals with SCI, only about half were satisfied with their bowel care routine because of the amount of time required, pain or discomfort, and generally unsatisfactory results, which were associated with reduced quality of life in the domains of bowel care, employment, and social function [3]. A safe and effective pharmacological approach to bowel care would be a welcome addition to clinical care for the individual with SCI and DWE.

Persons with SCI require at least one therapeutic intervention to initiate defecation, and most patients report bowel dysfunction as a major life-limiting problem. In one report, constipation (56%, 31/55) and incontinence (42%, 23/55) were the most common gastrointestinal problems. Digital rectal stimulation was the most common method for bowel evacuation, regardless of whether patients participated in a bowel program or not [11]. In a retrospective analysis of a cross-sectional phone survey of 64 patients determined which bowel management methods were evaluated and a Likert-type questionnaire was applied to assess the impact of neurogenic bowel disorders on both the International Classification of Function, Disability and Health (ICF) domains and on quality of life [4]. The most common bowel management methods were laxatives, suppositories and osmotic laxatives; of note, 50.1% of patients scored moderate or severe NBD. For reporting by patient for the ICF domains of Environmental and Personal factors, 46.9% had loss of privacy, 45.3% had a need of assistance for bowel management, 45.3% had feelings of frustration, anxiety or depression, and 39.1% reported neurogenic bowel to be associated with increased economics costs [4]. There was also a significant impact on the ICF category of Body Structures, with 26.6% of patients reporting complaints of pain associated with neurogenic bowel problems; for the ICF Activity domain, 28.1% reported an impact to achieve scheduled activities, 26.6% reported impact on the time spent in defecation, and 23.4% reported the need of diet adaptions [4]. A significant association was found between severity of neurogenic bowel disorder and a negative impact on quality of life (*p* < 0.05) [4]. Inskip et al. reported that management of bowel dysfunction was a problem for 78% of 287 individuals with SCI who were surveyed, and this condition proved to be a problem with personal relationships (60%), prevented from leaving home (62%), and interfered with employment outside the home (41%) [12]. In 24% of respondents with SCI, the routine bowel care regimen lasted longer than 60 min and most persons (59%) and required digital rectal stimulation to complete the bowel care session. Despite the best efforts of patients, bowel incontinence was reported at least monthly in 33% of those queried [12]. Autonomic dysreflexia due to bowel care interfered with activities of daily living in 51% of subjects. Longer durations of bowel care were highly significantly correlated with lower quality of life [12]. There are also economic costs associated with bowel dysfunction. In a survey of 332 patients with fecal incontinence for more than a year with at least monthly leakage of stool, the average annual total cost per person was $4110, with the severity of fecal incontinence correlated to higher annual direct costs [13].

The development of a successful bowel care routine in an individual with SCI is approached empirically. Medications that are employed in the management of bowel dysfunction may be divided by route of administration (e.g., by mouth or per rectum) or by their pharmacological mechanism of action. The categories of agents include bulk-forming agents, stool softeners, and laxatives. Various direct bowel wall stimulants have been used, and include senna preparations by mouth or per rectum, castor oil, magnesium preparations by mouth, and sodium phosphate/biphosphate by mouth. Even after applying these measures to induce a regular bowel evacuation, persons with SCI frequently have incomplete and unpredictable bowel evacuation, which may result in discomfort, autonomic dysreflexia, and/or stool incontinence. The inability to empty the colon predictably and completely results in a high risk of incontinence. Fecal incontinence is a source of hummiliation, lost time, increased caregiver support and additional expense. The possibility of bowel accidents is often provided as a reason that those with SCI remain homebound and have a tendency to avoid activities in the community. Prior to our work, few, if any, evidence-based pharmacologic interventions improve fecal transit time and bowel evacuation in a predictable manner in those with SCI. Trans-anal irrigation has been employed as a treatment to reduce constipation and fecal incontinence when other more conservative modalities prove unsatisfactory. When conservative treatments are not effective, surgical interventions may be considered.

The drug combination of neostigmine, a cholinergic agent, and glycopyrrolate, a selective cardiopulmonary anticholinergic agent, administered by intravenous [5], and intramuscular [6] route was demonstrated by our group to predictably stimulate bowel evacuation without life-threatening cholinergic effects on heart rate or airway [5,14]. The effectiveness and reliability of this dual pharmacological approach to induce bowel evacuation is appreciably greater than that of oral or rectal cathartics. However, the practical utility of prescribing agents by infusion for routine bowel care is limited because certified medical personnel are required to administer medications, as well as the inconvenience and risks of infection with intravenous drug delivery. In addition, the parenteral route of drug delivery often meets resistance from patients. Intramuscular delivery of agents, if administered above the level of lesion, would be painful and may be associated with pain and hematomas that may impair mobility and transfers and, if delivered below the level of lesion, may precipitate autonomic dysreflexia in those with higher cord lesions (e.g., above thoracic level-6). The transdermal administration of these agents by iontophoresis was identified as an alternative route of administration [8]. In three subjects with spinal cord lesions above thoracic level-6, each with a history of autonomic dysreflexia, Faaborg et al. reported that performing digital rectal evacuation or transanal irrigation resulted in substantial blood pressure elevations [15]. Five of the six subjects reported herein had higher spinal cord lesions and each had a history of intermittent autonomic dysreflexia; despite their histories of autonomic dysreflexia, the dual drug combination appeared to be at least as safe as the two bowel interventions reported by Faaborg et al., but additional work should be performed in a larger number of patients with higher cord lesions to confirm this finding. As reported in an earlier report, a 40% success rate was attained to induce bowel evacuation by transcutaneous administration of neostigmine and glycopyrrolate; however, when employing a modified protocol for the transcutaneous administration of these agents, the ability to induce a bowel evacuation was accomplished in each subject for all six of their bowel care sessions in the work reported herein.

The administration of the dual drug combination may result in untoward cholinergic or anti-cholinergic side-effects. Neostigmine may be safely administered by transcutaneous route when the ratio of neostigmine to glycopyrrolate is 5:1, which appears to antagonize the cholinergic effects of neostigmine on the heart and lungs to a clinically sufficient extent but spares the prokinetic effect of neostigmine on the bowel; our group is posed to further define the most clinically beneficial ratio of neostigmine to glycopyrrolate by transcutaneous administration to successfully induce bowel evacuation with the least adverse, albeit relatively minor, side effects. In our prior work with the intravenous administration of these agents, which had a greater frequency and intensity of minor adverse events, the major adverse cardiopulmonary manifestations of neostigmine (e.g., severe bradycardia and/or bronchoconstriction) did not occur [8].

This research has high, as well as immediate, translational potential to clinical care for persons with SCI who have difficulty with bowel evacuation. The work presented herein included only hospitalized patients who required greater than an hour for routine bowel care. Thus, it remains to be established if the transdermal delivery of neostigmine and glycopyrrolate has utility for those with shorter durations of bowel care in the home setting. However, the dual drug combination appears to have advantages over other bowel care approaches, including other prokinetic agents such as prucalopride [16], because the approach studied herein induces a prompt, predictable, and a more complete bowel evacuation. The dual drug combination may also be considered as an alternative approach for patients with severe neurogenic bowel following SCI who may otherwise be receiving transanal irrigation as their method for performing routine bowel care [17] or for those considering an intestinal diversion procedure [18]. However, the use of a relatively difficult to use, wired iontophoresis device for a person with SCI remains a major obstacle for this drug delivery approach to be transferred to routine clinical care. However, the development and commercialization of a wireless iontophoresis patch system that is user-friendly and is currently being developed would overcome this obstacle, allowing individuals with SCI to regain far greater control over bowel function than is possible with the bowel care regimens that are currently available. Such an advance in bowel care should markedly reduce the occurrence of complications due to constipation, stool impaction, stool incontinence, and anal pathologies. A far more successful approach to bowel care would permit those with SCI to regain a degree of independence, as well as prove useful as an adjunctive therapy for individuals with other disabilities who suffer from chronic constipation.

In summary, the addition of neostigmine and glycopyrrolate by transcutaneous administration to standard bowel care regimens in persons with SCI and DWE resulted in the safe, effective, and predictable bowel evacuation with subjective improvement in bowel care. The adverse events reported with this approach to bowel care were minor and transient.

## Figures and Tables

**Table 1:** Characteristics of the Study Participants.

						SOC and NEO + GLY Treatment		
Subject ID	Age (year)	TSI (year)	ISNCSCI (A/B/C/D)	MI (C/I)	SI (C/I)	Baseline Body Weight (kg)	Week-2 Body Weight (kg)	Body Weight △
001	62	3	B	C	I	96.4	93.8	−2.6
002	39	29	C	I	I	74.6	71.5	−3.1
003	54	8	D	I	I	77.8	73.4	−4.4
004	62	13	C	I	I	132	129.6	−2.4
005	66	47	B	C	I	61.4	60.0	−1.4
006	64	5	B	C	I	71.8	69.0	−2.8
Mean (SD)	57.8 (10.1)	17.5 (17.2)	0/3/2/1	3/3	0/6	85.7 (25.4)	82.9 (25.5) *	−2.8 (0.98)

Values are expressed for individual participants and as a group mean ± standard deviation (SD). Abbreviations: ISNCSCI = International Standards for Neurological Classification of Spinal Cord Injury; TSI = time since injury; kg = kilogram; MI = motor impairment; SI = sensory impairment; C = complete; I = incomplete; SOC = standard of care; NEO = neostigmine, GLY = glycopyrrolate; **△** = difference. * Baseline Body Weight vs. Week-2 Body Weight (post dual drug treatment): *p* < 0.001.

## Data Availability

Data presented are contained within the article.

## References

[1] Stiens S.A., Bergman S.B., Goetz L.L. (1997). Neurogenic bowel dysfunction after spinal cord injury: Clinical evaluation and rehabilitative management. Arch. Phys. Med. Rehabil..

[2] Krassioukov A., Eng J.J., Claxton G., Sakakibara B.M., Shum S. (2010). Neurogenic bowel management after spinal cord injury: A systematic review of the evidence. Spinal Cord.

[3] Pardee C., Bricker D., Rundquist J., MacRae C., Tebben C. (2012). Characteristics of neurogenic bowel in spinal cord injury and perceived quality of life. Rehabil. Nurs..

[4] Pires J.M., Ferreira A.M., Rocha F., Andrade L.G., Campos I., Margalho P., Laíns J. (2019). Assessment of neurogenic bowel dysfunction impact after spinal cord injury using the International Classification of Functioning, Disability and Health. Eur. J. Phys. Rehabil. Med..

[5] Korsten M.A., Rosman A.S., Ng A., Cavusoglu E., Spungen A.M., Radulovic M., Wecht J.M., Bauman W.A. (2005). Infusion of neostigmine-glycopyrrolate for bowel evacuation in persons with spinal cord injury. Am. J. Gastroenterol..

[6] Rosman A.S., Chaparala G., Monga A., Spungen A.M., Bauman W.A., Korsten M.A. (2008). Intramuscular neostigmine and glycopyrrolate safely accelerated bowel evacuation in patients with spinal cord injury and defecatory disorders. Dig. Dis. Sci..

[7] Kumar M., Chawla R., Goyal M. (2015). Topical anesthesia. J. Anaesthesiol. Clin. Pharmacol..

[8] Korsten M.A., Lyons B.L., Radulovic M., Cummings T.M., Sikka G., Singh K., Hobson J.C., Sabiev A., Spungen A.M., Bauman W.A. (2017). Delivery of neostigmine and glycopyrrolate by iontophoresis: A nonrandomized study in individuals with spinal cord injury. Spinal Cord.

[9] Lynch A.C., Wong C., Anthony A., Dobbs B.R., A Frizelle F. (2000). Bowel dysfunction following spinal cord injury: A description of bowel function in a spinal cord-injured population and comparison with age and gender matched controls. Spinal Cord.

[10] Atkinson M.J., Sinha A., Hass S.L., Colman S.S., Kumar R.N., Brod M., Rowland C.R. (2004). Validation of a general measure of treatment satisfaction, the Treatment Satisfaction Questionnaire for Medication (TSQM), using a national panel study of chronic disease. Health Qual. Life Outcomes.

[11] Ozisler Z., Koklu K., Ozel S., Unsal-Delialioglu S. (2015). Outcomes of bowel program in spinal cord injury patients with neurogenic bowel dysfunction. Neural Regen. Res..

[12] Inskip J.A., Lucci V.-E.M., McGrath M.S., Willms R., Claydon V.E. (2018). A Community Perspective on Bowel Management and Quality of Life after Spinal Cord Injury: The Influence of Autonomic Dysreflexia. J. Neurotrauma.

[13] Xu X., Menees S.B., Zochowski M.K., Fenner D.E. (2012). Economic cost of fecal incontinence. Dis. Colon Rectum.

[14] Radulovic M., Spungen A.M., Wecht J.M., Korsten M.A., Schilero G.J., Bauman W.A., Lesser M. (2004). Effects of neostigmine and glycopyrrolate on pulmonary resistance in spinal cord injury. J. Rehabil. Res. Dev..

[15] Faaborg P.M., Christensen P., Krassioukov A.V., Laurberg S., Frandsen E., Krogh K. (2014). Autonomic dysreflexia during bowel evacuation procedures and bladder filling in subjects with spinal cord injury. Spinal Cord.

[16] Krogh K., Jensen M.B., Gandrup P., Laurberg S., Nilsson J., Kerstens R., De Pauw M. (2002). Efficacy and tolerability of prucalopride in patients with constipation due to spinal cord injury. Scand. J. Gastroenterol..

[17] Christensen P., Bazzocchi G., Coggrave M., Abel R., Hultling C., Krogh K., Media S., Laurberg S. (2006). A randomized, controlled trial of transanal irrigation versus management in spinal cord-injured patients. Gastroenterology.

[18] Coggrave M., Ingram R., Gardner B., Norton C. (2012). The impact of stoma for bowel management after spinal cord injury. Spinal Cord.

